# Associations between Timing and Duration of Eating and Glucose Metabolism: A Nationally Representative Study in the U.S.

**DOI:** 10.3390/nu15030729

**Published:** 2023-02-01

**Authors:** Marriam Ali, Sirimon Reutrakul, Gregory Petersen, Kristen L. Knutson

**Affiliations:** 1 Center for Circadian and Sleep Medicine, Department of Neurology, Northwestern University Feinberg School of Medicine, 710 N Lakeshore Drive, Room 1003, Chicago, IL 60611, USA; 2 Department of Endocrinology and Metabolism, Loyola University Stritch School of Medicine, Maywood, IL 60153, USA; 3 Division of Endocrinology, Diabetes and Metabolism, Department of Medicine, University of Illinois Chicago, Chicago, IL 60607, USA

**Keywords:** diet, diabetes mellitus, insulin resistance, epidemiology, circadian rhythm

## Abstract

Diabetes is highly prevalent and is associated with dietary behaviors. Time-restricted eating, which consolidates caloric intake to a shortened eating duration, has demonstrated improvement in metabolic health. Timing of eating could also impact metabolism. Our objective was to examine whether the timing of eating was associated with metabolic health independently of eating duration. Data (n = 7619) are from four cycles (2005–2012) of the National Health and Nutrition Examination Survey (NHANES), a nationally representative U.S. survey that included surveys, physical examinations, and dietary recalls. The primary exposures are eating duration and eating start time estimated from two non-consecutive dietary recalls. Primary outcomes were fasting glucose and estimated insulin resistance using the homeostatic model assessment method (HOMA-IR). The mean (95% CI) eating duration was 12.0 h (11.9–12.0) and the mean (95% CI) start time was 8:21 (8:15–8:26). Earlier eating start time was significantly associated with lower fasting glucose and estimated insulin resistance but eating interval duration was not. Every hour later that eating commenced was associated with approximately 0.6% higher glucose level and 3% higher HOMA-IR (both *p* < 0.001). In this cross-sectional study, earlier eating start time was associated with more favorable metabolic measures, indicating that meal timing is another important characteristic of dietary patterns that may influence metabolism.

## 1. Introduction

The traditional approach to managing diabetes has focused on modifying insulin resistance and supplementing insulin through medications. Despite the emergence of newer pharmacologic therapies, metabolic disorders such as diabetes mellitus remain on the rise. The number of adults living with diabetes has more than tripled over the past 20 years, which surpassed previous global projections [1]. Research incorporating lifestyle interventions has typically centered on improving the amount and type of physical activity and modifying dietary intake. Although lifestyle interventions to increase physical activity and/or reduce caloric intake can reduce diabetes risk [2], they have not reduced diabetes prevalence at the population level since rates continue to rise. Therefore, there is an increased need for novel lifestyle interventions, including the study of dietary approaches as they relate to the circadian system and sleep-wake behavior. Thereby, the timing of food intake, which can influence the circadian system and metabolic regulation, has emerged as potentially important to metabolic health and possibly modifiable through behavioral interventions [3].

The circadian system is a set of endogenous molecular clocks that rhythmically govern various behavioral, physiological, and molecular changes over a 24-h period. A central clock located in the hypothalamus synchronizes with peripheral clocks in important metabolic organs including pancreatic β-cells, as well as with the environment to maintain metabolic homeostasis [3]. The 24-h circadian cycle is entrained or synchronized with the 24-h day through an environmental light signal to the central clock. The central clock then, in turn, synchronizes the peripheral clocks, which work in synchrony for metabolic advantage. For example, the circadian system was found to influence glucose tolerance such that glucose tolerance is greater in the morning than in the evening, and this is independent of any behaviors, such as physical activity or meals [4]. This diurnal pattern of glucose tolerance occurs through the pancreatic clock’s control of insulin release and the gut clock’s influence on intestinal glucose absorption. The circadian disruption hypothesis postulates that optimal metabolic health requires synchrony between these internal circadian clocks, sleep-wake and feeding patterns, and the hormones by which these rhythms and behaviors impact one another [3]. 

One metabolically relevant timing signal for synchronizing circadian clocks is feeding, which is a key synchronizer of peripheral clocks in the liver, gut, and muscle [5]. In the modern world, the rhythms of feeding behavior are often altered due to social demands, the constant availability of food, and extended access to light. Mistimed feeding can result in the desynchrony of circadian clocks, so-called “circadian disruption” or “circadian misalignment,” leading to metabolic dysfunction [5]. Eating later in the day during a weight loss program was shown to be associated with both lower insulin sensitivity and less weight loss compared to eating earlier, despite no differences in dietary intake [6,7]. Another study reported that eating dinner after 8 PM was associated with higher hemoglobin A1c than dinner consumption occurring before 8 PM [8]. In addition to the timing of feeding, evidence suggests that the duration of eating is also important. Time-restricted eating, or intermittent fasting, consolidates all caloric intake into a fixed and shortened eating duration, without directly altering total calorie intake or diet quality. Time-restricted eating has resulted in significant improvement in body weight, waist circumference, and blood pressure [9]. Similarly, pharmacological management with antihypertensive and lipid-lowering agents coupled with 10-h time-restricted eating was found to have beneficial effects on cardiovascular health measures in patients with metabolic syndrome [10], suggesting that these dietary timing interventions could act synergistically with medications.

As lifestyle modification, including diet, is increasingly recognized as a pillar of metabolic disease management, it is also necessary to refine our understanding of the best approaches to improve diet and, subsequently, metabolic health. For example, a recent 12-month study recognized that time-restricted eating to an 8-h duration was not more beneficial in reducing body weight than daily caloric restriction of 1500–1800 kcal/day for men and 1200–1500 kcal/day for women [11]. In addition, while feeding time and duration might have differential effects on metabolic health, they have rarely been considered together. This study sought to examine whether the timing of eating and eating duration were each independently associated with metabolic health in a large nationally representative cohort in the U.S. We hypothesized that eating at an earlier clock time and a shorter eating duration would each be associated with lower fasting glucose and estimated insulin resistance.

## 2. Materials & Methods

### 2.1. Sample

The sample includes men and women ≥ 20 years from the National Health and Nutrition Examination Survey (NHANES) study, which is a nationally representative study that included surveys and physical examinations and was conducted in 2-year waves [12]. NHANES used a four-stage sampling method to randomly select individuals for participation to compile a sample representative of the total noninstitutionalized civilian U.S. population. We combined four waves of data for these analyses: 2005–2006, 2007–2008, 2009–2010, and 2011–2012. All four waves used identical methodologies and instruments. 

### 2.2. Measurements

The measurements in these analyses come from dietary recalls, interviews, and physical examinations. NHANES collected two non-consecutive 24-h dietary recall interviews within a subset of participants. All NHANES participants were eligible for the dietary recall interviews. Participants could complete the dietary recall on any day of the week as long as they were not consecutive; 42% completed diaries on two weekdays (Monday through Friday), 54% completed diaries on one weekday and one weekend day, and 4% completed diaries on two weekend days. The dietary recall asked participants to report everything they consumed between midnight through 23:59 on the prior day. For these analyses, we set the behavioral day as beginning at 4:00 a.m. so that the earliest eating start time would be 4:00 a.m. Meals reported between midnight and 3:59 a.m. were considered evening meals. This time frame is similar to the time diaries used in the American Time Use Survey study that asks participants to report activities from 4:00 a.m. to 3:59 a.m. [13]. From the dietary recall, we calculated the time at first consumption (>0 kcal), which represents the eating interval start time. We also identified the time at last consumption (>0 kcal) and calculated the interval between the first and last consumption, which corresponds to the eating interval duration. Only participants with two dietary recall interviews are included in these analyses and we averaged these measures from the two dietary recalls. Total kilocalories (kcal) and nutrients consumed during the 24 h were estimated by NHANES. We excluded participants whose total caloric consumption during the 24-h period was deemed implausible, defined as <800 or >8000 kcal for men and <600 or >6000 for women [14]. The primary exposures in the analyses are eating interval duration and the timing of the first meal. In supplemental analyses, we examine associations between these timing measures and the following nutrients consumed over 24 h and averaged between the two recalls: protein (gm), total carbohydrates (g), total sugars (g), total fat (g), total saturated fatty acids (g), total monounsaturated fatty acids (g), total polyunsaturated fatty acids (g), and dietary fiber (g). 

The primary metabolic outcome measures were glucose and insulin assayed from the fasting blood sample obtained during the physical examination. All NHANES participants whose physical examination occurred in the morning were eligible. NHANES provides glucose in mg/dl and insulin in µU/mL, which we converted to SI units, mmol/L and pmol/L, respectively. We estimated insulin resistance using the homeostatic model assessment (HOMA-IR) formula [15]. 

Several covariates, obtained through the survey instruments, were included in the analyses. These measures were demographic information, such as age, gender (only male and female were provided as response options), self-identified race/ethnicity (Mexican American, non-Hispanic black, non-Hispanic white, other Hispanic or other race), smoking, alcohol use, diabetes, self-reported sleep duration, and education level. We also included body mass index (BMI), which was calculated using height and weight, measured during the physical examination by trained research staff.

### 2.3. Statistical Analysis

Our final sample size included 7619 participants. We estimated the unweighted sample means, standard deviations, and proportions for each variable as well as the weighted mean and proportion and their confidence intervals using the sample weights. We used ordinary least-squares linear regression models to estimate associations between the dietary timing measures (eating duration and eating start time) and the outcomes using sample weights (Stata/SE v14). We modeled the time variable in two ways: one model treated the eating interval and the start time as continuous variables simultaneously and the second model included categorical groups for these variables to test for nonlinear associations and were used in the figures. The eating interval groups were <10 h, 10–13 h (referent in models), and ≥13 h. Additionally, we also created 6 groups by dichotomizing sleep start time at 8:30 a.m. (referent was 10–13 h and ≤8:30 a.m.) in combination with eating interval groups. Models were adjusted for age, gender, BMI, race/ethnicity, education, diabetes, sleep duration, total kcal/day, smoking, alcohol use, and wave of data. Finally, we ran sensitivity analyses by excluding participants with diabetes to determine if associations persisted. Both glucose and HOMA-IR were log-transformed due to non-normal distribution. In secondary analyses, we examined the associations between the dietary timing measures (eating duration and eating start time) and the caloric and nutrient intake averaged between the two diary days. In these models we adjusted for the same covariates as above, except that total kcal/day was not included in Model 1 but was added in Model 2. 

## 3. Results

Ages ranged from 20–85 years (mean ± SD 49.3 ± 18.0) and approximately 50% were women. Table 1 describes the study sample, including both unadjusted values and values weighted to be nationally representative. The largest number of participants had an eating interval between 10–13 h and the average start time was approximately 8:30 a.m.

When examining eating interval duration and start time as continuous measures simultaneously, eating interval duration was not significantly associated with either fasting glucose or estimated insulin resistance (Table 2). However, eating start time was significantly associated with both. Every hour later that eating commenced was associated with approximately 0.6% higher glucose level and 3% higher HOMA-IR level (both *p* < 0.001). Results were identical when restricted to participants without diabetes (see Table S1).

**Figure 1:**
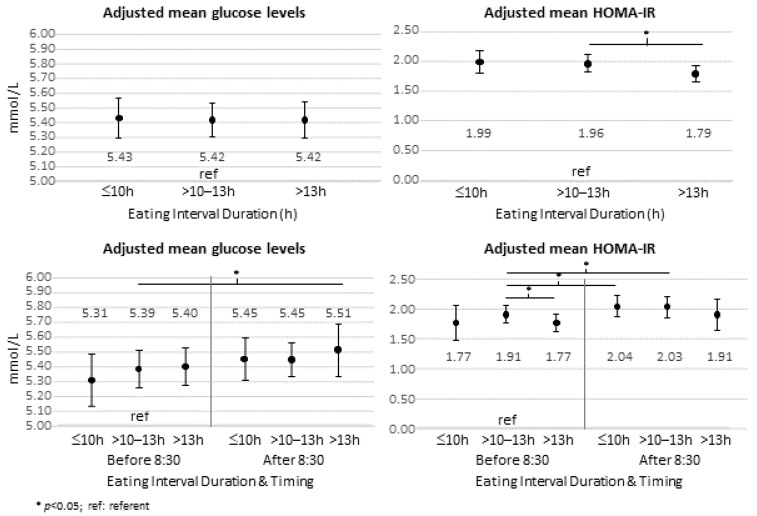
Adjusted mean glucose (**left**) and insulin resistance (HOMA-IR) (**right**) by eating interval duration (top two panels) and by eating interval groups categorized by start time at/before or after 8:30 a.m. (bottom two panels). Estimated means for each group are adjusted for age, gender, BMI, education level, race/ethnicity, total kcal/day, sleep duration, alcohol use, smoking, diabetes, and cohort and then back transformed.

Figure 1 presents the adjusted mean fasting glucose and HOMA-IR by eating interval duration groups and by eating interval groups divided by start time (at/before or after 8:30 a.m.). In adjusted regression models, fasting glucose did not differ significantly between eating interval groups, however, adjusted mean HOMA-IR was lowest in the longest eating interval duration group (*p* < 0.05). When divided by start time, adjusted mean fasting glucose level was higher in the group with the longest eating interval (>13 h) that started after 8:30 a.m. compared to those in the reference group (eating interval between 10–13 h and starting at or before 8:30 a.m.). The group with the longest eating interval that started at/before 8:30 a.m. had lower estimated insulin resistance compared to the referent, while the referent had lower insulin resistance than those with short (<10 h) or intermediate (10–13 h) eating duration that started later. 

We also examined whether the average caloric and nutrient intake varied by the eating duration groups as well as the eating duration groups divided by eating start time (Table 3). All of the nutrients varied by group; the longer eating duration group (>13 h) consumed more calories and greater amounts of the nutrients, including protein, carbohydrates, sugars, all types of fatty acids, and dietary fiber. 

When examining eating duration and start time as continuous variables (Table S2), a longer eating duration was significantly positively associated with caloric intake and all the nutrients. Similarly, a later start time was associated with greater caloric intake as well as greater intake of carbohydrates, sugars, total fat, and all fatty acids. Finally, since greater caloric intake will necessarily increase the number of nutrients consumed, we examined whether eating duration or start time was associated with the various nutrients after adjusting for total caloric intake (Table S2, lower half of table). A longer eating duration was associated with a greater intake of carbohydrates and sugars but a lower intake of fat, monounsaturated fatty acids, and polyunsaturated fatty acids after adjusting for caloric intake. A later eating start time was significantly associated with a lower intake of protein and dietary fiber but a greater intake of sugars and saturated fatty acids.

## 4. Discussion

Lifestyle modification has been recognized as a therapeutic foundation for combating metabolic disease. One of the approaches that have steadily gained attraction is time-restricted eating due to its relative ease of implementation and minimal complexity as well as its recognized benefits on markers of metabolic health. In this nationally-representative, cross-sectional analysis, we aimed to refine the metabolic underpinnings of time-restricted eating by analyzing eating clock time independently from eating duration. We observed that an earlier eating start time was significantly associated with lower fasting glucose and estimated insulin resistance, independently of eating interval duration and other covariates. However, a shorter eating interval duration was not significantly associated with lower fasting glucose levels and estimated insulin resistance, contrary to our hypotheses. Further, these associations remained similar after excluding people with diabetes.

Social alterations and technological advances have led to altered sleep-wake behaviors and meal patterns that subsequently mismatch with endogenous circadian rhythms, described as circadian misalignment. Erratic eating patterns with prolonged eating intervals have been observed in American adults [16]. Similarly, the majority of participants in our study had an eating window of 10–13 h, which is consistent with expanded eating intervals in the modern era. Studies have demonstrated that circadian misalignment leads to an increased risk of type 2 diabetes and other cardiometabolic diseases in healthy human volunteers [17] and particularly those who are shift workers [18]. Therefore, the concept of time-restricted eating or intermittent fasting emerged as a potential therapeutic intervention, and some prior research indeed identified shorter eating durations were associated with improved metabolic health [9].

We did not however find that shorter eating durations were associated with better metabolic outcomes, which is contrary to our own hypothesis and some prior work demonstrating that a shorter eating interval (i.e., intermittent fasting or time-restricted eating) was associated with better metabolic health [9,19]. Improvements in specific metabolic measures including insulin sensitivity and beta cell function were seen in time-restricted eating independently of weight loss in one study [20]. A recent study of 137 firefighters who worked 24-h shifts revealed that restricting eating duration to a 10 h window for 12 weeks led to a reduction in very low-density lipoprotein (VLDL) particle size as compared to usual eating duration [21]. Further, reductions in hemoglobin A1c and blood pressure were seen in those with elevated cardiometabolic risks at baseline [21]. Not all studies, however, have observed the beneficial effects of time-restricted eating. Another analysis of NHANES found similar results with respect to eating duration but did not test the independent effects of duration and timing on both glucose and insulin resistance [22]. More recently, a randomized control trial of time-restricted eating did not observe significantly greater weight loss compared to the control group [23], however, the time-restricted eating group did not begin eating until noon. Similarly, a randomized control trial of time-restricted eating did not observe significantly greater weight loss compared to standard dietary advice on weight, metabolic health, and processed foods [24]. Together, our study and these published studies highlight the need to further explore our understanding of time-restricted eating benefits, particularly with respect to clock time of eating.

Our findings on the timing of eating identified earlier clock times of meal intake are associated with beneficial metabolic outcomes, including lower fasting glucose levels and greater insulin sensitivity. Every hour later that eating commenced was associated with approximately 0.5% higher glucose level and 3% higher HOMA-IR level (both *p* < 0.001). Further, the difference in fasting glucose is about 0.06–0.16 mmol/L between the early eating groups and late eating groups. By comparison, the natural progression of fasting glucose in the general non-diabetic population is a rate of 0.04–0.06 mmol/L per 10 years of age [25]. Our findings on insulin sensitivity are consistent with prior studies that observed that eating earlier in the day was associated with beneficial metabolic outcomes, including insulin sensitivity [26,27,28]. In addition, a randomized cross-over study tested the impact of earlier timing of lunch (13:00) to a later lunch time (16:30) over one week and found that the earlier lunchtime was associated with greater glucose tolerance and greater fasting carbohydrate oxidation [29]. In another example, breakfast skipping was associated with impaired insulin response and increased post-prandial hyperglycemia at lunch and dinner [30]. Furthermore, in studies evaluating meal timing and weight loss, an earlier start time of meal intake was correlated with increased weight loss in participants undergoing a 12-week weight loss program and early time-restricted eating was more effective for weight loss than intake over 12 or more hours [27,31]. The underlying mechanisms for these associations likely involve the circadian system. There is a circadian rhythm of insulin sensitivity whereby insulin sensitivity is highest in the morning [32]. Further, the expressions of key hormones and proteins involved in metabolism are highest during the first five hours of the active phase (i.e., wake), and these include levels of corticosteroids, leptin, adiponectin, and PPAR-γ [33]. Therefore, eating earlier may be optimal because it coincides with greater insulin sensitivity and levels of these hormones and proteins, which together promote optimal metabolic function. Further, the timing of food intake is a powerful stimulus for circadian entrainment, and therefore eating at inopportune times, i.e., later in the day, has the potential to cause misalignment of metabolic processes [5]. Late and delayed eating is associated with weight gain and altered energy expenditure as well as abnormalities in appetite and stress hormones [34]. The beneficial effects of time-restricted eating, particularly earlier in the day, are yet to fully be determined.

We also examined whether caloric and nutrient intake was associated with these dietary timing measures. We found that a longer eating duration was associated with greater caloric intake as well as greater intake of all the nutrients. This may be simply due to eating over a longer period providing the opportunity to consume more. We also found that a later start time was associated with greater caloric intake and greater intake of carbohydrates, sugar, and fats. When adjusting for caloric intake, a later eating start time was associated with less healthy nutrient intake, i.e., more sugar and saturated fat but less protein and fiber. Carbohydrates and sugars, particularly ones with a higher glycemic index, have been linked to insulin action [35] and therefore diet quality could be one mechanism underlying the associations observed between later eating start time and higher fasting glucose and greater insulin resistance in this analysis. Our analyses are based on a large, national-representative study and examined both eating duration and timing simultaneously, which is a strength of this paper. However, a limitation is the cross-sectional design. An additional limitation is that the NHANES survey did not specifically identify shift workers and therefore we could not control for this potential confounder. Further, what was consumed the day prior to the blood sample is unknown. Nonetheless, these findings along with prior work indicate that the timing of food intake should be considered when developing and testing novel dietary interventions to improve metabolic health.

## 5. Conclusions

To our knowledge, the effect of meal timing independent of eating duration is under-explored, and our research aimed to differentiate these eating characteristics to assist future investigations focused specifically on dietary timing. In our study, an earlier eating start time was associated with better metabolic health and poor diet quality may play a role. Thus, future intervention studies of dietary patterns should consider the timing of meals in addition to the duration of eating as well as the composition of the diet.

## Figures and Tables

**Table 1:** Description of primary exposure and outcome measures (n = 7619).

	Unweighted Mean (SD) or No. (%)	Weighted Mean or % (95% CI) *
Age (years)	49.8 (17.9)	47.5 (46.7–48.2)
Women	3920 (51.5%)	51.9% (50.6–53.2)
Race and ethnicity		
Mexican American	1228 (16.1%)	7.6% (6.2–9.2)
Non-Hispanic Black	1466 (19.2%)	10.3% (8.7–12.1)
Non-Hispanic White	3755 (49.2%)	72.1% (68.8–75.1)
Other Hispanic	680 (8.9%)	4.6% (36–5.9)
Other Race	490 (6.4%)	5.5% (4.7–6.5)
Education level		
<9th grade	796 (10.5%)	5.4% (4.7–6.1)
9–11th grade	1144 (15.0%)	11.0% (9.8–12.5)
High school degree/GED	1776 (23.3%)	23.3% (21.5–25.2)
Some College or AA degree	2170 (28.5%)	30.6% (29.0–32.3)
College graduate or above	1733 (22.7%)	29.7% (27.3–32.3)
Current Smoker	1518 (19.9%)	20.0% (18.5–21.6)
Alcohol Use (drinks/week)	1.0 (1.8)	1.2 (1.1–1.3)
Self-reported sleep duration (hours)	6.8 (1.4)	6.9 (6.8–6.9)
BMI (kg/m^2^)	29.0 (6.7)	28.8 (28.6–29.1)
Underweight (BMI < 18.5 kg/m^2^)	112 (1.5%)	1.4% (1.1–1.8)
Ideal weight (BMI 18.5– < 25 kg/m^2^)	2095 (27.5%)	29.7% (28.0–31.4)
Overweight (BMI 25– < 30 kg/m^2^)	2596 (34.1%)	33.7% (32.3–35.0)
Obese (BMI ≥ 30 kg/m^2^)	2816 (37.0%)	35.3% (33.6–37.0)
Diabetes	1111 (14.5%)	10.8% (9.8–11.8)
Fasting Glucose (mmol/L)	6.0 (1.9)	5.8 (5.7–5.9)
HOMA-IR	3.8 (5.3)	3.4 (3.2–3.5)
Total kcal/day	2046 (788)	2118 (2090–2146)
Eating Interval Duration (h)	11.8 (2.2)	12.0 (11.9–12.0)
<10	1491 (19.6%)	18.0% (16.8–19.3)
10–13	4083 (53.6%)	52.1% (50.4–53.8)
>13	2045 (26.8%)	30.0% (28.5–31.4)
Eating Interval Start Time (hh:mm)	8:29 (1:52)	8:21 (8:15–8:26)
At or before 8:30	4536 (59.5%)	62.5% (60.1–64.8)
After 8:30	3083 (40.5%)	37.5% (35.2–40.0)
Eating Interval Duration + Start Time		
At/before 8:30 + <10 h	294 (3.9%)	3.3% (2.8–3.9)
At/before 8:30 + 10–13 h	2420 (31.8%)	32.4% (30.3–34.5)
At/before 8:30 + >13 h	1822 (23.9%)	26.8% (25.3–28.4)
After 8:30 + <10 h	1197 (15.7%)	14.7% (13.5–16.0)
After 8:30 + 10–13 h	1663 (21.8%)	19.7% (18.4–21.2)
After 8:30 + >13 h	223 (2.9%)	3.1% (2.7–3.7)

* Weighted mean uses sample weights to approximate nationally representative estimates.

**Table 2:** Results from regression models examining associations between fasting glucose, HOMA-IR, and eating duration and start time ^a^.

	(ln) Fasting Glucose		(ln) HOMA-IR	
	Regression Coeff	95% CI	Regression Coeff	95% CI
Eating Duration (hours)	0.002	−0.001, 0.005	−0.005	−0.017, 0.007
Eating Start Time (hours)	**0.006**	**0.003, 0.008**	**0.030**	**0.016, 0.044**

^a^ Models are adjusted for age, gender, BMI, race/ethnicity, education, diabetes, sleep duration, total kcal/day, smoking, alcohol use, and a wave of data. Bold indicates *p* < 0.05.

**Table 3:** Weighted means for 2-day average kcal and nutrient intake by eating duration groups and by eating duration groups split at mean start time.

	Total Kcal	Protein (g)	Total Carbohydrates (g)	Total Sugars (g)	Total Fat (g)	Total Saturated Fatty Acids (g)	Total Mono-Unsaturated Fatty Acids (g)	Total Poly-Unsaturated Fatty Acids (g)	Dietary Fiber (g)
**Eating Duration Groups**									
<10 h	1846.1 *	74.1 *	225.2 *	97.9 *	70.1 *	22.3 *	25.4 *	15.5 *	14.2 *
10–13 h (ref)	2086.4	82.3	253.6	111.6	79.0	25.8	28.6	17.7	17.2
>13 h	2332.3 *	91.2 *	280.8 *	128.7 *	89.1 *	29.5 *	32.4 *	19.6 *	18.5 *
**Eating Duration + Start Time Groups**									
At/before 8:30 and:									
<10 h	1747.5 *	72.0 *	215.9 *	92.2 *	64.9 *	21.3 *	23.5 *	14.2 *	16.3
10–13 h (ref)	2044.5	82.2	245.9	106.7	78.0	25.3	28.3	17.6	17.4
>13 h	2306.8 *	90.8 *	277.6 *	127.2 *	88.3 *	29.2 *	32.1 *	19.4 *	18.5 *
After 8:30 and:									
<10 h	1868.5 *	74.6 *	227.3 *	99.1 *	71.2 *	22.6 *	25.9 *	15.8 *	13.8 *
10–13 h	2156.0	82.4	266.4 *	119.7 *	80.8	26.7 *	29.2	17.9	16.9
>13 h	2562.5 *	95.3 *	309.0 *	142.7 *	96.5 *	32.4 *	34.8 *	21.1 *	18.3 *

* *p* < 0.05 compared to the referent (ref).

## Data Availability

All data are publicly available at https://www.cdc.gov/nchs/nhanes/index.htm (accessed on 30 January 2023).

## Supplementary Materials

The following supporting material can be downloaded at https://www.mdpi.com/article/10.3390/nu15030729/s1, Table S1: Results from regression models examining associations between fasting glucose, HOMA-IR, and eating duration and start time excluding respondents with diabetes; Table S2: Results from regression models examining associations between eating duration and start time and caloric and nutrient intake.
