# Supplementary material for: Associations between Timing and Duration of Eating and Glucose Metabolism: A Nationally Representative Study in the U.S
Source: Nutrients. 2023 Feb 1;15(3):729. doi: 10.3390/nu15030729 (PMC9919634; doi:10.3390/nu15030729)
Supplement: Supplementary file 1 [file nutrients-15-00729-s001.zip › nutrients-2104838-supplementary.pdf]

Supplemental Material for

Title: Associations between Timing and Duration of Eating and Glucose Metabolism: A Nationally Representative Study in the U.S.

Authors: Marriam Ali, MD, Sirimon Reutrakul, MD, Gregory Petersen, PhD, Kristen L. Knutson, PhD

Journal: *Nutrients*

This document contains the following supplemental material:

1. Results from regression analyses examining associations between dietary timing variables and fasting glucose and HOMA-IR excluding respondents with diabetes.
2. Results from regression analyses examining associations between dietary timing variables and caloric and nutrient intake.

Table S1: Results from regression models examining associations between fasting glucose, HOMA-IR and eating duration and start time excluding respondents with diabetes (n=7,299).

	(ln) Fasting Glucose		(ln) HOMA-IR	
	Regression Coeff	95% CI	Regression Coeff	95% CI
Eating Duration (hours)	.002	-.001, .004	-.001	-.013, .011
Eating Start Time (hours)	**.005**	**.003, .007**	**.028**	**.012, .043**

[a] Models are adjusted for age, gender, BMI, race/ethnicity, education, sleep duration, total kcal/day, smoking, alcohol use, and wave of data.

Bold indicates $p<.05$.

Table S2: Results from regression models examining associations between eating duration and start time and caloric and nutrient intake (n=7,619).

	Total Kcal	Protein (g)	Total carbohydrates (g)	Total sugars (g)	Total fat (g)	Total saturated fatty acids (g)	Total monounsaturated fatty acids (g)	Total polyunsaturated fatty acids (g)	Dietary fiber (g)
	Reg Coeff (95% CI)	Reg Coeff (95% CI)	Reg Coeff (95% CI)	Reg Coeff (95% CI)	Reg Coeff (95% CI)	Reg Coeff (95% CI)	Reg Coeff (95% CI)	Reg Coeff (95% CI)	Reg Coeff (95% CI)
Model 1[a]									
Eating Duration (h)	**104.7 (91.3 – 118.2)**	**3.1 (2.3 – 3.8)**	**13.0 (11.1 – 15.0)**	**7.6 (6.3 – 8.9)**	**4.1 (3.4 – 4.7)**	**1.5 (1.3 – 1.8)**	**1.5 (1.2 – 1.7)**	**0.8 (0.7 – 1.0)**	**0.5 (0.3 – 0.8)**
Eating Start Time (h)	**44.3 (29.4 – 59.2)**	0.45 (-0.37 – 1.26)	**5.9 (3.7 – 8.2)**	**3.9 (2.5 – 5.4)**	**1.9 (1.2 – 2.6)**	**0.8 (0.5 – 1.0)**	**0.6 (0.4 – 0.9)**	**0.3 (0.1 – 0.5)**	-0.1 (-0.4 - 0.1)
Model 2[b]									
Eating Duration (h)		-0.4 (-0.9 – 0.1)	**1.1 (0.8 – 2.1)**	**2.0 (0.9 – 3.0)**	**-0.4 (-0.8 – -0.04)**	0.05 (-0.1 – 0.2)	**-0.2 (-0.4 – -0.03)**	**-0.1 (-0.3 – -.005)**	-0.1 (-0.3 – 0.1)
Eating Start Time (h)		**-1.0 (-1.6 – -0.5)**	0.9 (-0.3 – 2.1)	**1.5 (0.4 – 2.7)**	0.02 (-0.4 – 0.5)	**0.1 (-0.1 – 0.3)**	-0.1 (-0.2 – 0.1)	-0.1 (-0.2 – 0.1)	**-0.4 (-0.7 – -.02)**

[a] Model 1 is adjusted for age, gender, BMI, race/ethnicity, education, diabetes, sleep duration, total kcal/day, smoking, alcohol use, and wave of data.
[b] Model 2 adjusts for Model 1 covariates + total kcal.
Bold indicates p<.05.
